# Bulleyaconitine A Inhibits Visceral Nociception and Spinal Synaptic Plasticity through Stimulation of Microglial Release of Dynorphin A

**DOI:** 10.1155/2020/1484087

**Published:** 2020-05-23

**Authors:** Sheng-Nan Huang, Jinbao Wei, Lan-Ting Huang, Pei-Jun Ju, Jinghong Chen, Yong-Xiang Wang

**Affiliations:** ^1^Shanghai Key Laboratory of Psychotic Disorders, Shanghai Mental Health Center, Shanghai Jiao Tong University School of Medicine, Shanghai, China; ^2^King's Lab, School of Pharmacy, Shanghai Jiao Tong University, Shanghai, China

## Abstract

**Background:**

Visceral pain is one of the most common types of pain and particularly in the abdomen is associated with gastrointestinal diseases. Bulleyaconitine A (BAA), isolated from *Aconitum bulleyanum*, is prescribed in China to treat chronic pain. The present study is aimed at evaluating the mechanisms underlying BAA visceral antinociception.

**Methods:**

The rat model of chronic visceral hypersensitivity was set up by colonic perfusion of 2,4,6-trinitrobenzene sulfonic acid (TNBS) on postnatal day 10 with coapplication of heterotypic intermittent chronic stress (HeICS).

**Results:**

The rat model of chronic visceral hypersensitivity exhibited remarkable abdominal withdrawal responses and mechanical hyperalgesia in hind paws, which were dose-dependently attenuated by single subcutaneous of administration of BAA (30 and 90 *μ*g/kg). Pretreatment with the microglial inhibitor minocycline, dynorphin A antiserum, and *κ*-opioid receptor antagonist totally blocked BAA-induced visceral antinociception and mechanical antihyperalgesia. Spontaneous excitatory postsynaptic currents (sEPSCs) in spinal dorsal horn lamina II neurons were recorded by using whole-cell patch clamp. Its frequency (but not amplitude) from TNBS-treated rats was remarkably higher than that from naïve rats. BAA (1 *μ*M) significantly reduced the frequency of sEPSCs from TNBS-treated rats but not naïve rats. BAA-inhibited spinal synaptic plasticity was blocked by minocycline, the dynorphin A antiserum, and *κ*-opioid receptor antagonist. Dynorphin A also inhibited spinal synaptic plasticity in a *κ*-opioid receptor-dependent manner.

**Conclusions:**

These results suggest that BAA produces visceral antinociception by stimulating spinal microglial release of dynorphin A, which activates presynaptic *κ*-opioid receptors in afferent neurons and inhibits spinal synaptic plasticity, highlighting a novel interaction mode between microglia and neurons.

## 1. Introduction

Visceral pain refers to pain caused by noxious stimuli to activate nociceptors in the internal organs such as those in the chest and abdomen. It is one of the most common types of pain in clinic and particularly in the abdomen is often associated with gastrointestinal diseases, such as irritable bowel syndrome, functional dyspepsia, and inflammatory bowel disease. Colonic inflammation in patients sensitizes primary afferent neurons to cause visceral pain [1]. In addition, stress in life can also induce visceral hypersensitivity or pain [2–4]. Currently, chronic visceral pain is still hard to be treated clinically [5–7]. As a result, investigators are committed to searching for more effective means to alleviate visceral pain. Bulleyaconitine A (BAA) is a diterpenoid alkaloid isolated from *Aconitum bulleyanum*, belonging to the “aconitine-like” alkaloids. In the formulations of tablets, intramuscular injections, and soft gel capsules, it has been used for treatment of chronic pain since it was approved in 1985 in China [8, 9]. BAA exhibits somatic antinociception in many rodent models of painful hypersensitivity, including formalin-induced tonic hyperalgesia [9], spinal nerve ligation- and paclitaxel-induced neuropathic pain, and bone cancer pain [10, 11]. However, the antinociceptive effect of BAA on visceral pain has not yet been systemically investigated.

Various proposals have been postulated for the mechanisms underlying the antinociceptive effects of BAA or its analogs. Voltage-dependent sodium (Nav) channels, such as Nav1.7, Nav1.8, and Nav1.9 channels, are promising target molecules for the treatment of neuropathic pain and other chronic pain [12–15]. Interactions with Nav channels were extensively reported to be associated with the antinociceptive effects of BAA and its analogs. Previous studies showed that the state-dependent blockade of Nav1.7 and Nav1.8 channels in afferent neurons has been suggested to contribute to BAA-induced long-term cutaneous antinociception as an adjuvant in rats [8]. The latest research showed that BAA attenuated hyperexcitability of dorsal root ganglion neurons induced by spared nerve injury by the use-dependent blockade of tetrodotoxin-sensitive Nav1.3 and Nav1.7 channels [11]. In addition, it was proposed that activation of the descending inhibitory transmission by norepinephrine and serotonin was associated with the antinociceptive effects of BAA and its analogs mesaconitine, 3-acetylaconitine, and lappaconitine [9, 16, 17]. In contrast, our laboratory recently demonstrated that BAA and its analogs aconitine, bullatine, and lappaconitine, given subcutaneously or intrathecally, blocked the spinal nerve ligation-induced neuropathic pain by the stimulation of the spinal microglial expression of dynorphin A [10, 18–20].

The current study is aimed at evaluating the antinociceptive effect of BAA in the model of visceral hypersensitivity and testing the hypothesis that the mechanism underlying BAA visceral antinociception was through stimulating the spinal microglial extension of dynorphin A. We first set up a rat model of colonic hypersensitivity by colonic perfusion of TNBS (2,4,6-trinitrobenzene sulfonic acid), followed by application of heterotypic intermittent chronic stress (HeICS), and then tested the visceral antinociceptive effect of BAA. Later on, the microglial inhibitor minocycline, dynorphin A antiserum, and *κ*-opioid receptor antagonist nor-NBI were employed to intervene BAA antinociception. Moreover, BAA was reported to inhibit C-fiber-stimulated long-term potential and enhanced spinal synaptic transmission from paclitaxel-induced neuropathic rats [21]. We postulated that the dynorphin A released from microglia would activate presynaptic *κ*-opioid receptors of afferent neurons leading to inhibition of spinal synaptic plasticity and central sensitization. Thus lastly, the recording of spontaneous excitatory postsynaptic currents (sEPSCs) of spinal dorsal horn, by using whole-cell patch clamp technique, was set up from TNBS-treated rats to test the possible inhibitory effect of BAA on spinal synaptic plasticity.

## 2. Material and Methods

### 2.1. Drugs and Reagents

Bulleyaconitine A (BAA), minocycline, and nor-binaltorphimine dihydrochloride (nor-BNI) were purchased from Zelang Bio-Pharmaceutical (Nanjing, China), Yuanye Biotech (Shanghai, China), and Abcam (Cambridge, United Kingdom), respectively. TNBS (2,4,6-trinitrobenzene sulfonic acid) and 5′-guanidinonaltrindole (5′-GNTI) were obtained from Sigma-Aldrich (St. Louis, MO, USA). Dynorphin A (1–17) of YGGFLRRIRPKLKWDNQ was synthesized by Dan Gang Peptides Co. (Hangzhou, China) with its purity not less than 98%. The rabbit dynorphin A antiserum was purchased from Phoenix Pharmaceuticals (Burlingame, CA, USA), with its specificity to dynorphin A (100%), but not to dynorphin B (0%), *β*-endorphin (0%), *α*-neo-endorphin (0%), or leu-enkephalin (0%), according to the manufacturer's description. Its specificity was also validated by the antigen absorption test from other laboratories [22, 23]. All of the reagents and drugs were diluted or dissolved in 0.9% normal saline or artificial cerebrospinal fluid (ACSF) except for TNBS which was dissolved in the 10% ethanol/90% saline solution.

### 2.2. Animals

Female Sprague-Dawley pup rats with a nurturing mother were obtained from Qianbi Biotechnology Company (Shanghai, China). These pup rats were weaned at 22 days of age and then housed three per cage. The animals were housed in a controlled environment (12/12 hr light-dark cycle, 23°C), receiving water and food *ad libitum*. The researchers were blinded for the behavior tests. The research protocols were approved by the Experimental Animal Committee of Shanghai Jiao Tong University School of Medicine following the Animal Care Guidelines of National Institutes of Health.

### 2.3. Induction of Neonatal Colonic Inflammation

To induce neonatal inflammation, TNBS (130 mg/kg, equaling to 2.86 mg for the approximately 22-gram pups, dissolved in 200 *μ*l saline containing 10% ethanol) was injected intraluminally 2 cm into the colon of female pups on postnatal day 10. The neonatal pup rats were mildly anesthetized with 2% isoflurane during colonic perfusion. The pups in the control group were perfused with the same volume of saline. For preventing leakage from the colons, the pups were put in a head down posture and the anus were closed for approximately 2 minutes [24, 25].

### 2.4. Heterotypic Intermittent Chronic Stress (HeICS)

The HeICS protocol which involved daily and multiple stressors was proven to induce anxiety-like behaviors and visceral hypersensitivity in the adult rats [26–30]. Four randomly arranged types of stress experiments, i.e., water avoidance stress, cold restraint stress, forced swimming stress, and electricity foot shock, in a variable schedule for consecutive 2 weeks were applied to rats previously subjected to the TNBS or to saline rats. The four kinds of stress experiments were circled to the same rat in sequence and a stress experiment was conducted in the morning and afternoon, respectively. The control group rats were kept undisturbed except for changing cages. For the water avoidance stress, the rats were placed for 2 hours on a cylinder (6 cm diameter × 15 cm high) as an island in the middle of the plastic container (60 × 60 × 60 cm) filled with water within 2-3 cm from the top. For the forced swimming stress, the rats were forced to swim for 20 minutes in a plastic container (21 cm diameter × 55 cm high) filled to a 25 cm depth below the top of the container with water at a temperature of 25 ± 1°C. For the cold restraint stress, the rats were restrained in a transparent plastic container (5 diameter × 10 cm long) with 10~15 holes to ensure normal breathing. The restraining container was placed in a refrigerator at 4°C for 45 minutes. For the electricity foot shock, the rats were placed in the fear conditioning box (15 high × 16 wide × 20 cm long), and the electricity shock current was 0.5 mA continuously for 10 seconds, repeating 30 times with an interval of 1 minute.

### 2.5. The Rat Intrathecal Injection

The intrathecal injection method was undertaken as described previously [31, 32]. In brief, rats were anesthetized with 2% isoflurane and the lumbar region of the back and lateral surface of the left thigh was shaved. A 50 *μ*l Hamilton syringe attached with a 27-gauge needle was inserted between the L5 and L6 vertebra until the intrathecal space was reached as indicated by tail twitch. The drug or the vehicle was injected in a volume of 10 *μ*l.

### 2.6. Evaluation of Visceromotor Response to Graded Colorectal Distention

The visceral hypersensitivity was evaluated by grading the response to colorectal distention as described previously [33]. For compliance, the balloons (7-8 mm diameter) were inflated overnight to stretch the latex the day before the abdominal withdrawal reflex evaluation. Rats, fasted 24 hours earlier, were anesthetized with isoflurane and the balloon coated with glycerinum was inserted into the colon and fixed with the tail 2-3 cm from the anus. The balloon was connected to the desktop sphygmomanometer through a T-branch pipe. Rats were given 30 minutes to accommodate the environment after they woke up. The balloon was then distended at four different pressures (20, 40, 60, and 80 mmHg) of graded colorectal distension. The distention at each pressure was maintained for 20 seconds at an interval of 5 minutes and repeated for three times. After colorectal dilatation, the rats gradually exhibited abdominal withdrawal responses at each pressure. The rating criteria of abdominal withdrawal reflex were as follows: 0, rats had no significant changes in behaviors; 1, rats did not move or only had simple head movements; 2, the abdominal muscles began to contract; 3, the low abdominal walls were lifted off the bottom of the box or significantly contracted and flattened; and 4, the abdominal walls were accompanied the bodies and pelvises were arched or the testicles were lifted.

### 2.7. Test of Mechanical Hyperalgesia

To evaluate mechanical hyperalgesia, the rats were brought into the testing room and placed in a plexiglass box, which was on a metal grid (0.5 × 0.5 cm). Then rats were adapted to the test environment for at least 30 minutes. The hind paw withdrawal thresholds were measured using a 2450 CE Electronic Von Frey hair (IITC Life Science, Woodland Hill, CA, USA). An electronic hand-held transducer with a number 15 monofilament was used perpendicularly to the medial surface of hind paws with a gradually increasing force (ranging from 0.1 to 90 g) until the rats suddenly licked or withdrew their hind paws. The lowest force that produced a withdrawal response was recorded automatically and considered as the paw withdrawal threshold. Three repeated measurements were made at each time-point with an interval of approximately 3 minutes, and their threshold values were averaged.

### 2.8. Spinal Cord Slice Preparation

The methods to obtain the rat spinal cord slice preparations were described previously [34]. In brief, the 4-week-old rats were deeply anesthetized and the L4-L6 lumbar enlargements were quickly moved to the preoxygenated and ice-cold-modified ACSF which contained (in mM) 80 NaCl, 2.5 KCl, 1.25 NaH_2_PO_4_, 0.5 CaCl_2_, 3.5 MgCl_2_, 25 NaHCO_3_, 75 sucrose, 1.3 sodium ascorbate, and 3.5 sodium pyruvate, with pH 7.4 and osmolality of 310–320 mOsm. After opening the dura and cutting the dorsal root, the spinal cord was cut into 400 *μ*m slices on a VT1000S vibratome (Leica, Germany). Slices were then incubated for about 1 hour at 35°C in a preoxygenated solution containing (in mM) 125 NaCl, 2.5 KCl, 2 CaCl_2_, 1 MgCl_2_, 1.25 NaH_2_PO_4_, 26 NaHCO_3_, 25 D-glucose, 1.3 sodium ascorbate, and 3.0 sodium pyruvate, with a pH of 7.2 and measured osmolality of 310–320 mOsm. The spinal cord slices were then transferred into a submerged recording chamber and perfused with the preoxygenated recording solution at 3 ml/min before whole-cell recording.

### 2.9. Whole-Cell Patch Clamp Recording

The whole-cell recording was conducted in spinal dorsal horn neurons with a glass microelectrode, which was made from thin-walled, glass-capillary tubings (1.0 mm OD, 0.5 mm ID; Sutter Instruments, Novato, CA, USA) with a horizontal puller (P-87, Sutter Instruments). Their osmolality was 7-12 M*Ω* when they were filled with the pipette solution containing the following (in mM): 130 potassium gluconate, 4 Na_2_ATP, 5 KCl, 20 HEPES, 0.5 NaGTP, and 0.5 EGTA, with a pH of 7.3 and measured osmolality of 310-320 mOsm. After a gigaseal formation (seal resistance: 2–50 G*Ω*), the membrane was ruptured to obtain the whole-cell voltage clamp configuration.

For the sEPSC recording, 10 *μ*M bicuculline and 2 *μ*M strychnine were added to the bath solution to prevent the inhibitory responses. Spinal dorsal horn neurons in lamina II were voltage clamped at -70 mV. Series resistances for all spinal dorsal horn neurons recorded in this research were within 30 M*Ω*. Data were obtained and analyzed by using the Axoclamp 200B amplifier and pCLAMP software (Axon Instruments, Foster City, CA, USA). The drugs were both applied by exchanging a perfusion solution which contained a known drug concentration.

### 2.10. Statistical Analyses

All data are presented as mean ± standard error of mean (SEM). A one-way and repeated-measures two-way ANOVA followed by Fisher's post hoc analysis was used for comparison of means of each group. *P* < 0.05 was considered statistically significant for the differences.

## 3. Results

### 3.1. TNBS Perfusion and HeICS Application-Induced Visceral Nociception and Mechanical Allodynia

**Figure 1:**
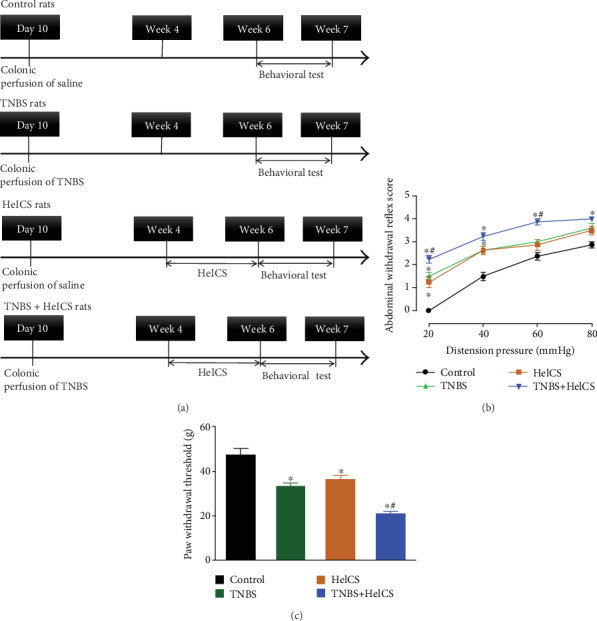
Timeline of the visceral hypersensitivity experimental protocols (a). Abdominal withdrawal reflexes to graded colorectal distention (b) and hind paw withdrawal thresholds to mechanical stimuli (c) in control-, TNBS-, HeICS-, and TNBS+HeICS-treated rats. The visceral hypersensitivity protocols included colonic perfusion of TNBS on postnatal day 10, and 4 weeks later followed by two weeks of HeICS, which included water avoidance stress, cold restraint stress, forced swimming stress, and electricity foot shock. The data are presented as means ± SEM (*n* = 8 in each group). ^∗^ and ^#^ denote *P* < 0.05 compared to the saline control and TNBS, HeICS, or TNBS+HeICS treatment, respectively, by one-way or repeated-measures two-way ANOVA followed by Fisher's post hoc analysis.

Four groups of pup rats were subjected to colonic perfusion of normal saline or TNBS colonic inflammation on postnatal day 10, and 4 weeks later followed by two weeks of HeICS, which included water avoidance stress, cold restraint stress, forced swimming stress, and electricity foot shock (Figure 1(a)). The abdominal withdrawal reflexes to graded colorectal distention and hind paw withdrawal thresholds to mechanical stimuli were measured after the last time of HeICS application. As shown in Figure 1(b), colorectal distension by balloon inflation induced abdominal withdrawal responses in a pressure-dependent manner (20-80 mmHg). Either perfusion with TNBS or application of HeICS significantly lifted colorectal distension-induced pressure-response curve, compared to the control rats (*P* < 0.05 by the repeated-measures two-way ANOVA followed by Fisher's post hoc analysis). More significantly, double treatments with TNBS perfusion and subsequently HeICS application exacerbated abdominal hypersensitivity scores, which were greater than those of either TNBS treatment or HeICS application alone (*P* < 0.05 by repeated-measures two-way ANOVA followed by Fisher's post hoc analysis). In addition, hind paw withdrawal thresholds were measured by using electric Von Frey hairs. TNBS treatment and HeICS application each significantly reduced paw withdrawal thresholds compared to the control rats. More significantly, double treatments with TNBS perfusion and HeICS application reduced hind paw withdrawal thresholds, which were lower than those of TNBS perfusion or HeICS application alone (*P* < 0.05 by one-way ANOVA followed by Fisher's post hoc analysis). Thus, the rats doubly challenged with TNBS perfusion and HeICS application were later used to test the antinociceptive effects of BAA.

### 3.2. Subcutaneous Administration of BAA Produced Visceral Antinociception

**Figure 2:**
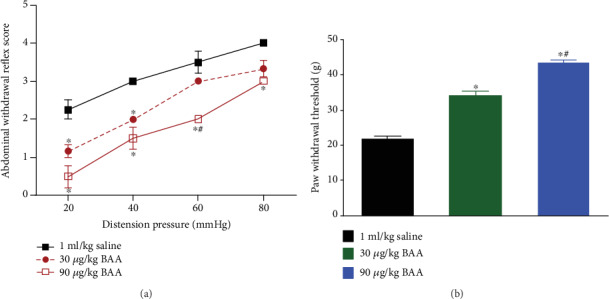
The dose-dependent inhibitory effect of bulleyaconitine (BAA, 30 and 90 *μ*g/kg), given subcutaneously, on TNBS perfusion+HeICS application-induced visceral hypersensitivity (a) and mechanical hyperalgesia (b) in rats. The visceral hypersensitivity protocols included colonic perfusion of TNBS on postnatal day 10 and 4 weeks later followed by two weeks of HeICS, which included water avoidance stress, cold restraint stress, forced swimming, and electricity foot shock. The data are presented as means ± SEM (*n* = 8 in each group). ^∗^ and ^#^ denote *P* < 0.05 compared to the normal saline control and TNBS+HeICS treatment, respectively, by one-way or repeated-measures two-way ANOVA followed by Fisher's post hoc analysis.

Three groups of TNBS+HeICS-treated rats received a single subcutaneous injection of normal saline (1 ml/kg) or BAA (30 or 90 *μ*g/kg). Based on our previous finding that BAA in neuropathic rats exhibited peak antihypersensitivity effect at 1 hour after its subcutaneous administration [10], the visceral antinociceptive effect of BAA was evaluated 1 hour after its injection. As shown in Figure 2(a), colorectal distension by balloon inflation induced pressure-dependent abdominal withdrawal hypersensitivity in saline-treated rats. Subcutaneous injection of BAA (30 and 90 *μ*g/kg) dose-dependently lowered the pressure-response curve (*P* < 0.05 by repeated-measures two-way ANOVA followed by Fisher's post hoc analysis). Similarly, the mechanical antihyperalgesic effect of BAA was also measured 1 hour after injection. Double treatments with TNBS+HeICS in hind paws produced mechanical hyperalgesia, which was dose-dependently reduced by BAA injection (*P* < 0.05 by one-way ANOVA followed by Fisher's post hoc analysis; Figure 2(b)).

### 3.3. BAA-Induced Visceral Antinociception Was through Stimulation of Spinal Microglial Expression of Dynorphin A

**Figure 3:**
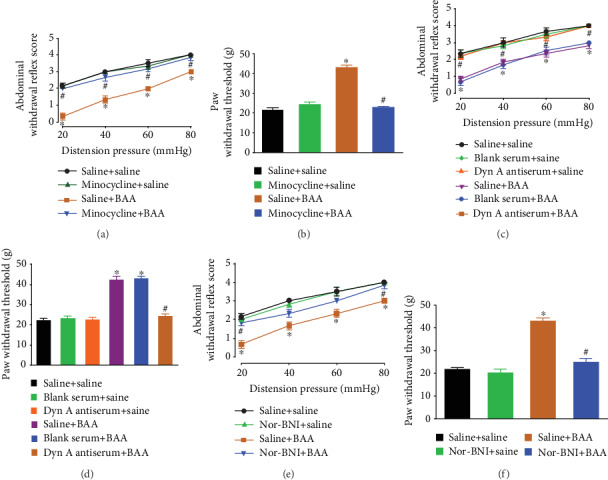
The blockade effects of subcutaneous, the microglial inhibitor minocycline (30 mg/kg (a, b)), intrathecal, the dynorphin A antiserum (1 : 10 dilution, 10 *μ*l (c, d)), and subcutaneous, the *κ*-opioid receptor antagonist nor-BNI (10 mg/kg (e, f)), on subcutaneous injection of bulleyaconitine- (BAA-, 90 *μ*g/kg) induced visceral antinociception and mechanical antihyperalgesia in TNBS+HeICS-treated rats. The visceral hypersensitivity protocols included colonic perfusion of TNBS on postnatal day 10 and 4 weeks later followed by two weeks of HeICS, which included water avoidance stress, cold restraint stress, forced swimming stress, and electricity foot shock. The data are presented as means ± SEM (*n* = 6 in each group). ^∗^ and ^#^ denote *P* < 0.05 compared to the normal saline control and TNBS+HeICS treatment, respectively, by one-way or repeated-measures two-way ANOVA followed by Fisher's post hoc analysis.

BAA and its analogs bullatine and lappaconitine have been demonstrated to specifically stimulate dynorphin A expression in spinal microglia [10, 18]. To test whether BAA produced visceral antinociception in a microglia-dependent manner, the microglia inhibitor minocycline [35, 36] was applied. Four groups of TNBS+HeICS-pretreated rats received two treatments after the last stress application. These two treatments were intraperitoneal injection of normal saline (1 ml/kg) or minocycline (30 mg/kg) followed 2 hours later by subcutaneous injection of saline (1 ml/kg) or BAA (90 *μ*g/kg). The abdominal and hind paw behavior tests were undertaken 1 hour after the last injection. Compared to the saline-treated rats, subcutaneous injection of BAA inhibited TNBS+HeICS-induced abdominal withdrawal responses. Although intraperitoneal injection of minocycline did not significantly affect baseline abdominal withdrawal responses, its pretreatment completely blocked BAA-induced visceral antinociception in the abdomen (*P* < 0.05 by repeated-measures two-way ANOVA followed by Fisher's post hoc analysis; Figure 3(a)). The inhibitory effect of minocycline was also confirmed in BAA-induced mechanical antihyperalgesia in hind paws (*P* < 0.05 by one-way ANOVA followed by Fisher's post hoc analysis; Figure 3(b)).

To test whether BAA produced visceral antinociception through stimulation of the expression of dynorphin A, the dynorphin A antiserum [10, 18] was applied. Six groups of TNBS+HeICS-pretreated rats received two treatments after the last stress application. These two treatments were intrathecal injection of saline (10 *μ*l), blank rabbit serum (1 : 10 dilution, 10 *μ*l), or dynorphin A antiserum (1 : 10 dilution, 10 *μ*l), and followed 0.5 hours later by subcutaneous injection of saline (1 ml/kg) or BAA (90 *μ*g/kg). The abdominal and hind paw behavior tests were undertaken 1 hour after the last injection. Compared to the saline control, subcutaneous injection of BAA produced significant visceral antihypersensitivity effect. Intrathecal injection of blank serum or the dynorphin A antiserum did not significantly affect baseline visceral hypersensitivity thresholds. However, the pretreatment with intrathecal injection of the dynorphin A antiserum (but not blank serum) completely inhibited subcutaneous injection of BAA-induced visceral antinociception in the abdomen (*P* < 0.05 by repeated-measures two-way ANOVA followed by Fisher's post hoc analysis; Figure 3(c)). The complete inhibitory effect of the dynorphin A antiserum was also confirmed in BAA-induced mechanical antiallodynia in hind paws (*P* < 0.05 by one-way ANOVA followed by Fisher's post hoc analysis; Figure 3(d)).

Dynorphin A is known to produce antinociception by acting on *κ*-opioid receptors [37–39]. Thus, we further tested whether BAA also exerted visceral antihypersensitivity effect through *κ*-opioid receptors by using the *κ*-opioid receptor antagonist nor-BNI [40]. Four groups of TNBS+HeICS-pretreated rats received two treatments after the last stress application. These two treatments were subcutaneous injection of normal saline (1 ml/kg) or nor-BNI (10 mg/kg), and followed 2 hours later by subcutaneous injection of saline (1 ml/kg) or BAA (90 *μ*g/kg). The abdominal and hind paw behavior tests were undertaken 1 hour after the last injection. As shown in Figures 3(e) and 3(f), subcutaneous injection of nor-BNI did not significantly affect baseline visceral hypersensitivity scores or hind paw mechanical hyperalgesia in TNBS+HeICS-treated rats. However, its pretreatment completely blocked BAA-induced visceral antinociception in the abdomen and mechanical antihyperalgesia in hind paws (*P* < 0.05 by one-way or repeated-measures two-way ANOVA followed by Fisher's post hoc analysis).

### 3.4. BAA Reduced TNBS-Enhanced Synaptic Transmission in Spinal Dorsal Horn Neurons

**Figure 4:**
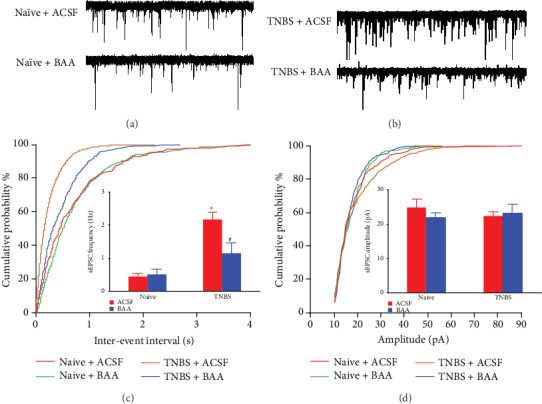
The effects of bulleyaconitine A (BAA, 1 *μ*M) on representative traces of spontaneous excitatory postsynaptic currents (sEPSCs) (a, b) and summarized frequency (c) and amplitude (d) of sEPSCs in spinal dorsal horn lamina II neurons from naïve and TNBS-treated rats. The visceral hypersensitivity protocol included colonic perfusion of TNBS on postnatal day 10. Four weeks later, the rats were killed and the spinal cords were removed for the sEPSC recording by whole-cell patch clamp. The data are presented as means ± SEM (*n* = 12 in each group). ^∗^ and ^#^ denote *P* < 0.05 compared to the naïve+ACSF group and TNBS+ACSF group, respectively, by one-way ANOVA followed by Fisher's post hoc analysis.

As shown in Figure 1(c) and even in our previous studies, the rats in TNBS treatment group showed an obvious and excellent performance in visceral pain phenotype, compared to the control group. Moreover, sEPSCs in spinal dorsal horn lamina II neurons were recorded during 4 weeks. Hence, to simplify the modeling method in subsequently recording the sEPSCs, we compared spinal dorsal horn synaptic transmission between naïve rats and TNBS-pretreated rats. Two groups of pup rats received colonic perfusion of normal saline and TNBS, respectively, on the tenth day of birth. Eighteen days later, the rats were killed and the spinal enlargements were removed. The sEPSCs were recorded in dorsal horn lamina II neurons by using the whole-cell patch clamp. As shown in the representative traces in Figures 4(a) and 4(b) and further analysis using the pCLAMP software in Figures 4(c) and 4(d), the frequency of sEPSCs in dorsal horn neurons from TNBS-treated rats was significantly higher than that from the naïve rats (*P* < 0.05 by one-way ANOVA followed by Fisher's post hoc analysis). However, there were no significant changes in the amplitude of sEPSCs between naïve and TNBS-treated rats. The results indicate an enhanced spinal synaptic transmission or synaptic plasticity was established in the visceral hypersensitivity state induced by TNBS.

Furthermore, the inhibitory effect of BAA, dissolved in the ACSF perfusion solution, on sEPSCs was tested. As shown in Figure 4, perfusion of 1 *μ*M BAA for 10 minutes significantly reduced the frequency of sEPSCs in dorsal horn neurons from TNBS-treated rats (*P* < 0.05 by one-way ANOVA followed by Fisher's post hoc analysis). However, BAA failed to alter the frequency of sEPSCs in dorsal horn neurons from naïve rats. In contrast, treatment with BAA did not significantly change the amplitude of sEPSCs either from naive rats or from TNBS-treated rats.

### 3.5. Minocycline, the Dynorphin A Antiserum, and 5′-GNTI Blocked BAA- and Dynorphin A-Induced Inhibition of Spinal Synaptic Plasticity

**Figure 5:**
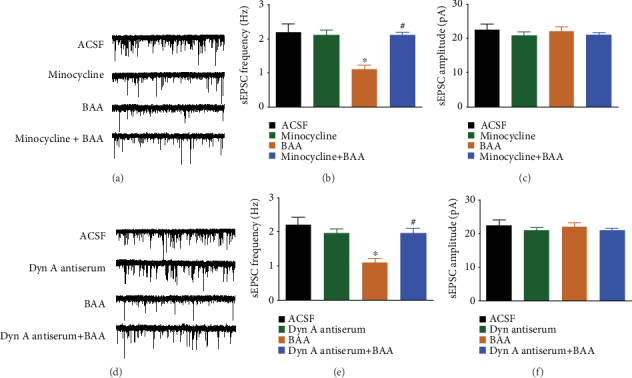
The blockade effects of the microglial inhibitor minocycline (1 *μ*M (a–c)), and dynorphin antiserum (1 : 50 dilution (d–f)) on bulleyaconitine A- (BAA-, 1 *μ*M) inhibited enhanced spontaneous excitatory postsynaptic currents (sEPSCs) in dorsal horn lamina II neurons from TNBS-treated rats. (a, d) Representative traces of sEPSCs. The visceral hypersensitivity protocol included colonic perfusion of TNBS on postnatal day 10. Four weeks later, the rats were killed and the spinal cords were removed for the sEPSC recording by whole-cell patch clamp. The data are presented as means ± SEM (*n* = 8 − 12 in each group). ^∗^ and ^#^ denote *P* < 0.05 compared to the ACSF group and BAA treatment group, respectively, by one-way ANOVA followed by Fisher's post hoc analysis.

To confirm the causal role of the microglial expression of dynorphin A in BAA-induced inhibition of spinal synaptic plasticity, the microglial inhibitor minocycline and the dynorphin A antiserum were employed in the sEPSC recording assay. As shown in Figures 5(a)–5(c), perfusion with 1 *μ*M of BAA for 10 minutes significantly reduced the frequency of sEPSCs without changing the amplitude. However, pretreatment (30 minutes earlier) with perfusion of minocycline (10 *μ*M) [41], dissolved in ACSF perfusion solution, completely blocked BAA-inhibited enhanced frequency of sEPSCs in spinal dorsal horn neurons from TNBS-treated rats (*P* < 0.05 by one-way ANOVA followed by Fisher's post hoc analysis), although it did not significantly alter the baseline values of enhanced synaptic transmission. Similarly, perfusion of the dynorphin A antiserum (1 : 50 dilution), dissolved in the ACSF perfusion solution, did not significantly alter baseline synaptic transmission. However, its pretreatment (30 minutes earlier) totally reversed BAA-induced inhibition of enhanced frequency of sEPSCs in spinal dorsal horn neurons from TNBS-treated rats (*P* < 0.05 by one-way ANOVA followed by Fisher's post hoc analysis) but did not significantly affect the amplitude of sEPSCs (Figures 5(d)–5(f)).

**Figure 6:**
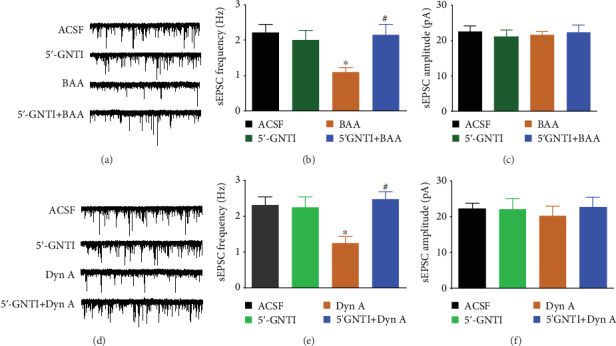
The blockade effects of the *κ*-opioid receptor antagonist 5′-GNTI (1 *μ*M) on bulleyaconitine A- (BAA-, 1 *μ*M (a–c)) and dynorphin A- (Dyn A-, 1 *μ*M (d–f)) inhibited enhanced spontaneous excitatory postsynaptic currents (sEPSCs) in dorsal horn lamina II neurons from TNBS-treated rats. (a, d) Representative traces of sEPSCs. The visceral hypersensitivity protocol included colonic perfusion of TNBS on postnatal day 10. Four weeks later, the rats were killed and the spinal cords were removed for the sEPSC recording by whole-cell patch clamp. The data are presented as means ± SEM (*n* = 12 in each group). ^∗^ and ^#^ denote *P* < 0.05 compared to the ACSF group and BAA or Dyn A treatment group, respectively, by one-way ANOVA followed by Fisher's post hoc analysis.

To further explore the association between activation of *κ*-opioid receptors and BAA-inhibited enhanced spinal synaptic transmission, the blockade effects of the specific *κ*-opioid receptor antagonist 5′-GNTI [42, 43] were employed. As exhibited in Figures 6(a)–6(c), pretreatment (30 minutes earlier) with bath application of 5′-GNTI (1 *μ*M), dissolved in the ACSF perfusion solution, totally blocked BAA-inhibited enhanced frequency (but not amplitude) of sEPSCs in spinal dorsal horn neurons from TNBS-treated rats (*P* < 0.05 by one-way ANOVA followed by Fisher's post hoc analysis), although it did not significantly alter baseline synaptic transmission. Moreover, perfusion with exogenous dynorphin A (1 *μ*M) [44] for 15 minutes in spinal dorsal horn neurons significantly inhibited enhanced frequency (but not amplitude) of sEPSCs, which was entirely blocked by pretreatment (30 minutes earlier) with perfusion of 5′-GNTI (1 *μ*M) (*P* < 0.05 by one-way ANOVA followed by Fisher's post hoc analysis; Figures 6(d)–6(f)).

## 4. Discussion

Early adverse experience could change neurological and endocrinological responses to stress later in life, which is manifested as enhanced responses to visceral pain and cutaneous stimulation [45–47]. There is an inevitable relationship between the surface reaction of human body and the changes of internal organs. The previous studies also proved that visceral lesions do have corresponding reactions on the body surface, such as visceral pain, accompanied by radiation pain on the body surface, i.e., referred pain. There is a kind of cross talk between different tissues and organs. The autonomic nerves of the viscera may be mixed with the pain nerves in a certain section of the spinal cord, resulting in visceral lesions which are often reflected in the pain of the surface area responsible for the spinal cord, which is mentioned to as “referred pain.” Visceral lesions are often accompanied by referred pain, and organic visceral pain can often find a more fixed skin sensitive zone. Hyperalgesia can also be interpreted as the formation of afferent nerve fibers from diseased visceral organs and involved body parts, which enter the spinal cord from the same posterior root and converge to the same neurons in the thalamic tract of the spinal cord. Hence, besides the visceromotor response to graded colorectal distention, hind paw mechanical hyperalgesia is mostly adopted to assess chronic hypersensitivity in animal models. Rats in our current study received TNBS perfusion on postnatal day 10, and HeICS application later developed remarkable abdominal cutaneous hyperalgesia and hind paw mechanical hyperalgesia. In this model of chronic visceral hypersensitivity, subcutaneous administration of BAA dose-dependently reduced abdominal withdrawal responses and mechanical hyperalgesia in hind paws. The results indicate that BAA produces visceral antinociception in the model of chronic visceral hypersensitivity induced by the combination of TNBS+HeICS treatments and expand its analgesic spectrum. BAA and its analogs aconitine, bullatine, and lappaconitine have been extensively reported to exhibit antinociception in various somatic painful hypersensitivity models, such as spinal nerve ligation- and paclitaxel-induced neuropathic pain, formalin- and complete Freund's adjuvant-induced inflammatory pain, bone cancer pain, and diabetic pain [10, 18, 19, 21]. It should be noted that, although we did not test it in the current study, BAA and its analogs have been shown to have slight or little inhibitory effects on normal pain thresholds in naïve rats or in the contralateral hind paws of unilateral peripheral nerve injury-induced neuropathic rats [10, 11, 21]. All the results taken together suggest that aconitum alkaloids particularly BAA produce specific antinociception in both somatic and visceral hypersensitivity states.

We have further demonstrated that BAA produced visceral antinociception by stimulation of spinal microglial expression of dynorphin A. This conclusion is supported by the following findings. (1) Our previous study revealed that BAA and its analogs aconitine, bullatine, and lappaconitine specifically stimulated the expression of dynorphin A in primary cultures of microglia but not of astrocytes or neurons from neonatal and adult rats, even though the latter two types of cells also expressed and secreted dynorphin A. Moreover, intrathecal injection of aconitines particularly BAA specifically stimulated microglial (but not astrocytic or neuronal) expression of dynorphin A in both the contralateral and ipsilateral spinal cords of neuropathic rats [10, 18, 19, 48]. (2) The visceral antinociceptive effect of BAA, given subcutaneously, was completely blocked by the pretreatment with intraperitoneal injection of the microglia inhibitor minocycline. (3) Particularly, the pretreatment with intrathecal injection of dynorphin A antiserum entirely attenuated subcutaneous BAA-induced visceral antinociception. (4) Dynorphin A is known to be an endogenous ligand of *κ*-opioid receptors [49, 50]. Pretreatment with subcutaneous injection of the *κ*-opioid receptor antagonist nor-BNI totally blocked BAA-induced visceral antinociception. In addition, BAA-induced mechanical antihyperalgesia in the hind paws in the rat model of visceral hypersensitivity was also attenuated by application of minocycline, the dynorphin A antiserum, and nor-BNI, same as that in spinal nerve ligation-induced neuropathic rats [10]. All these results reveal that spinal microglial expression of dynorphin A mediates both visceral and somatic antihypersensitivity effects.

Central sensitization is a key character in the pathology of chronic pain disorders, which refers to increased synaptic efficacy or synaptic plasticity in afferent neurons in the spinal dorsal horn following nerve damage, peripheral noxious stimulation, or tissue injury or inflammation [51–54]. The prolonged synaptic plasticity after a noxious stimulus is a key electrophysiological basis of central sensitization, which is majorly related to the release of neurotransmitter glutamate and activation of N-methyl-D-aspartate (NMDA) receptors in postsynaptic neurons [52, 54, 55]. The increased synaptic transmission causes the decrease in the pain threshold, expansion of pain responses, and spread of pain sensitivity to noninjured areas. Clinically, central sensitization leads to pain hypersensitivity in the viscera, skin, and muscle. Maintained central sensitization is an important mechanism underlying chronic pain including persistent inflammatory and neuropathic pain [55, 56]. We demonstrated that the frequency (but not amplitude) of sEPSCs in dorsal horn lamina II neurons from TNBS-treated rats was significantly higher than that from the naïve rats, suggesting that neonatal visceral inflammation induced by TNBS establishes spinal synaptic plasticity and central sensitization, which is consistent with the previous report in which colonic inflammation leads to central sensitization [54, 57, 58].

Consistent with the finding that BAA specifically inhibited enhanced sEPSCs or mEPSCs in dorsal horn lamina II neurons from paclitaxel-induced neuropathic rats (Zhu et al. [21]), our study demonstrated that BAA significantly reduced synaptic plasticity in spinal dorsal horn neurons in TNBS-treated rats but did not significantly affect synaptic transmission in naïve rats. The results indicate that BAA specifically inhibits spinal synaptic plasticity and central sensitization, which is probably the basis for BAA to specifically attenuate the visceral nociception in the abdomen and mechanical hyperalgesia in hind paws induced by the combination of TNBS and HeICS application. We further demonstrated that BAA reduced spinal synaptic plasticity through stimulation of microglial expression of dynorphin A, as perfusion of minocycline, the dynorphin A antiserum, and specific *κ*-opioid receptor antagonist 5′-GNTI totally blocked BAA-inhibited enhanced spinal synaptic transmission. Dynorphin A is an endogenous opioid neurotransmitter and can be produced in the spinal cord and many parts of the brain such as the striatum, hippocampus, and hypothalamus [59]. It acts through presynaptic *κ*-opioid receptors, reducing Ca^2+^-dependent glutamate secretion thereby inhibiting synaptic transmission and reducing neural plasticity in the hippocampus, the hypothalamic arcuate nucleus, the posterior paraventricular nucleus of the thalamus, and other nucleus [60–63]. In addition, the *κ*-opioid receptor agonist dynorphin A and U69593 in spinal neurons exert inhibitory effects on spontaneous calcium oscillations driven primarily by glutamatergic neurotransmission, which is completely blocked by the selective *κ* receptor antagonist nor-BNI. Consistent with the presynaptic mechanism of action reported for *κ*-opioid receptors, the expression of the *κ*-opioid receptor is enriched in spinal glutamatergic neurons with a relative abundance in the presynaptic compartment [64]. Indeed, we confirmed that dynorphin A inhibited enhanced presynaptic glutamate transmission in spinal dorsal horn lamina II neurons in a *κ*-opioid receptor-dependent manner.

**Figure 7:**
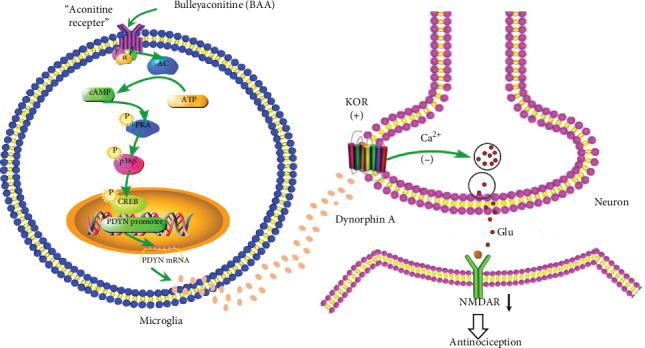
Schematic illustration of the spinal microglial dynorphin A/*κ*-opioid receptor/synaptic plasticity pathway for bulleyaconitine A (BAA) to produce antinociception in visceral hypersensitivity state. BAA stimulates spinal microglia via a Gs/cAMP/PKA/p38*β*/CREB signaling transduction pathway to express and secret dynorphin A, which passes through the microglial neuronal synapse and acts on the presynaptic *κ*-opioid receptors. Following presynaptic *κ*-opioid receptor activation, the enhanced neuronal presynaptic glutamate transmission and consequent postsynaptic NMDA currents are reduced in the visceral hypersensitivity state, which leads to inhibition of central sensitization and visceral antinociception.

Taken together, our results illustrate a specific mode of the cross talk between microglia and neurons in the spinal dorsal horn for BAA to produce visceral antinociception, i.e., BAA stimulates microglia to express and secret dynorphin A via a Gs/cAMP/PKA/p38*β*/CREB signal transduction mechanism [48], which passes through the microglial neuronal synapse and acts on the presynaptic *κ*-opioid receptor in afferent neurons. Following the activation of presynaptic *κ*-opioid receptors, the enhanced neuronal synaptic glutamate transmission and consequent postsynaptic NMDA currents are reduced in the visceral hypersensitivity state, leading to inhibition of central sensitization and antinociception. The illustration of the antinociceptive pathway of BBA in the visceral hypersensitivity is presented in Figure 7.

## Data Availability

The data used to support the findings of this study are available from the corresponding author upon request.

## Abbreviations

ACSF: Artificial cerebrospinal fluid
BAA: Bulleyaconitine A
HeICS: Heterotypic intermittent chronic stress
Nav: Voltage-dependent sodium
sEPSC: Spontaneous excitatory postsynaptic current
TNBS: 2,4,6-trinitrobenzene sulfonic acid.
